# Early change in left atrial function in patients treated with anthracyclines assessed by real-time three-dimensional echocardiography

**DOI:** 10.1038/srep25512

**Published:** 2016-05-05

**Authors:** Jing Shi, Ye Guo, Leilei Cheng, Feiyan Song, Xianhong Shu

**Affiliations:** 1 Department of Echocardiography, Zhongshan Hospital of Fudan University, Shanghai Institute of Cardiovascular Diseases, Shanghai Institute of Medical Imaging, Shanghai, China; 2 Department of Medical Oncology, Fudan University Shanghai Cancer Center, Shanghai, China

## Abstract

Real-time three-dimensional echocardiography(RT-3DE) has allowed a better assessment of LA volumes and function. We sought to assess the early change in left atrial size and function in patients treated with anthracyclines using RT-3DE. 61 patients aged 44.9 ± 11.9 years with large B-cell non-Hodgkin lymphoma treated with doxorubicin were studied. Blood collection and echocardiography were performed at baseline and 1 day after completion of the chemotherapy. Global longitudinal strain (GLS), maximum, minimum and pre-atrial contraction LA volumes were measured and reservoir, conduit and booster pump function were assessed. Despite normal LVEF, passive emptying percent of total emptying (0.51 ± 0.14 vs. 0.40 ± 0.12, *P* < 0.001) and passive emptying index (0.29 ± 0.10 vs. 0.23 ± 0.06, *P* < 0.001) were remarkably reduced compared to baseline values, while active emptying percent of total emptying (0.49 ± 0.14 vs. 0.60 ± 0.12, *P* < 0.001) and active emptying index (0.41 ± 0.16 vs. 0.47 ± 0.16, *P* = 0.048) were increased. GLS (−21.64 ± 2.83 vs. −17.30 ± 2.50) was markedly reduced, cTnT levels was elevated from 0.005 ± 0.004 to 0.020 ± 0.026 ng/mL at the completion of chemotherapy (*P* all  < 0.001). Early LA functional change occur after doxorubicin exposure in patients with preserved LVEF, which could be detected by RT-3DE.

Anthracyclines chemotherapy remains a critical component of cancer treatment despite its established risk of cardiotoxicity. It has been suggested that diastolic changes precede systolic dysfunction in various conditions and predict later deterioration^1^,^2^,^3^. Several studies have reported LV diastolic abnormalities late after anthracycline administration; these abnormalities were associated with wall motion abnormalities despite a preserved LVEF^4^,^5^. LA size and function is associated with LV diastolic function^6^. In fact, quantification of LA function remains chanllenging. Real-time three-dimensional echocardiography (RT3DE) has provided information about real time changes of LA volumes during cardiac cycle, allowing an easier and more reliable calculation of LA emptying fractions^7^,^8^,^9^. Recently, RT3DE has shown to be more accurate than 2DE (two-dimensional echocardiography) for the assessment of LA volumes, when both were compared with magnetic resonance^10^.

In our study we sought to assess the early change in left atrial size and function in patients treated with anthracyclines using three-dimensional echocardiography.

## Methods

### Study population

A total of 66 patients with newly diagnosed, histopathologically confirmed large B-cell non-Hodgkin lymphoma in fudan university shanghai cancer center were enrolled in this study between March 2012 and May 2015. Age < 18 years, left ventricular ejection fraction (LVEF) <50%, valvular heart disease, severe hypertension, life expectancy ≤12 weeks, arrhythmia, a previous history of heart failure and/or coronary artery disease were exclusion criteria for enrollment. All patients were treated with R-CHOP (cyclophosphamide 750 mg/m2, vincristine 1.4 mg/m2 up to a maximum dose of 2 mg, doxorubicin 50 mg/m2 on day 1, prednisone 100 mg on day 1–5), and rituximab 375 mg/m2. All therapy was given intravenously, except for prednisone, which was administered orally. Cycles were administered every 21 days for a maximum of 8 cycles. Rituximab was administered before chemotherapy. Patients were restaged every 2 cycles, and those with disease progression were withdrawn from the treatment of R-CHOP. No patients received other cardiotoxic agents or radiation therapy during this study. All patients gave informed consent, and the Fudan University Shanghai Cancer Center and Zhongshan Hospital Research Ethics Committee approved the protocol. Our study were carried out in accordance with the approved guidelines from the ethics committee.

### Echocardiographic imaging

A standard 2DE and RT3DE examination were performed for all patients. All 2DE and RT3DE examinations were performed with a commercially available ultrasound system (iE33, Phlips Medical Systems, N.A., Bothell, WA, USA) equipped with an S5-1 and X3-1 transducer. Measurements were made off line in a dedicated workstation (QLAB version9.0 for PC; Philips Medical System, N.A., Bothell, WA, USA).

LV ejection fraction was calculated by means of 2DE, using modified biplane Simpson’s rule. Conventional spectral Doppler variables (mitral E and A diastolic waves, DT: E deceleration time, IVRT: isovolumetric relaxation time) and tissue Doppler data (mitral annular diastolic e’ and a’ waves) were obtained according to standard guidelines^11^,^12^.

The automatic tracking analysis was performed in the apical four-chamber, two-chamber, apical LV longitudinal view for longitudinal strain by two-dimensional speckle tracking analysis.

**Figure 1:**
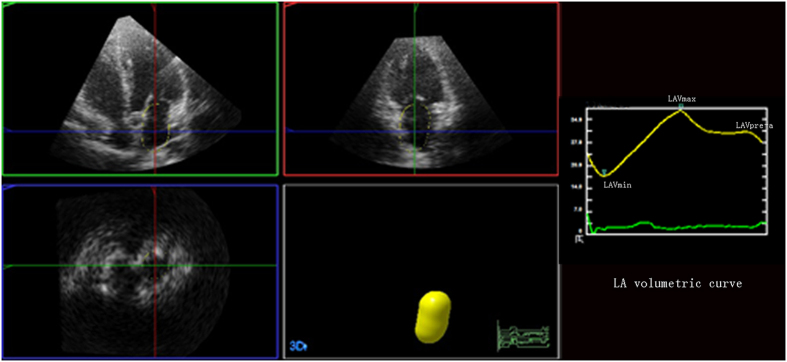
Example of one three-dimensional echocardiography study. LAVmax (maximum left atrial volume), LAVmin (minimal left atrial volume), LAVpre-a(preatrial left atrial volume).

In order to analyze LA volumes and function by RT3DE, an apical full-volume dataset was acquired combining 4 electrocardiogram-triggered subvolumes, within breath hold. A semiautomated contour-tracing software, designed for LV volume analysis (3DQ adv, Philips Medical System), was used to obtain LA volumes and volumetric curves. This is performed by marking 5 points in the atrial surfaces of the mitral annulus: at anterior, inferior, lateral and septal annuli and the 5^th^ point at apex of LA. Once this is completed, the endocardial surface was automatically delineated and a mathematical model of the LA calculation was obtained. Manual modification was made to correct the automatic tracing if needed (Fig. 1).

The following LA volumes were calculated: maximum atrial volume (LAVmax), the largest atrial volume at end systole; minimal atrial volume (LAVmin), at end diastole; volume before atrial contraction (LAVpre-a), the atrial volume in the last frame before or at time of P-wave. All comparisons between LA volumes were made considering volumes indexed to body surface area. LA reservoir function was assessed by 3 indices. Filling volume was calculated by LAVmax-LAVmin. Expansion index was analyzed by ([LAVmax-LAVmin]/LAVmin) × 100. Diastolic emptying index was determined by ([LAVmax-LAVmin]/LAVmax) × 100. LA conduit function was assessed by calculating the passive emptying percent of total emptying (PE) as ([LAVmax-LAVpre-a]/[LAVmax-LAVmin] × 100 and the passive emptying index (PEI) as ([LAVmax-LAVpre-a]/LAVmax) × 100. Booster pump function was assessed as follows. Active emptying percent of total emptying (AE) was calculated as ([LAVpre-a-LAVmin]/[LAVmax-LAVmin]) × 100. Active emptying index (AEI) was assessed by ([LAVpre-a-LAVmin]/LAVpre-a) × 100^13^.

All the recorded images were analyzed at different times by two independent echocardiographers. The same images were also analyzed on a different day by one of these same observers.

### High sensitivity cardiac troponin T assays

Blood was collected into EDTA tubes, centrifuged, and the plasma was removed and stored at −80 °C. The cTnT concentrations were measured with highly sensitive cTnT reagents on an Elecysys 2010 analyser (Roche Diagnostics, Indianapolis, IN, USA), with a lower detection limit of 0.003 ng/mL and a reported 99^th^ percentile value in apparently healthy individuals of 0.014 ng/mL^14^. Technologists recording the cTnT results were blind to the clinical and echocardiographic data of participants.

### Reproducibility

Intra- and inter-observer reproducibility was assessed by calculating the difference between the values of 10 randomly selected patients measured by 1 observer twice and by a second observer.

### Statistical analysis

Continuous variables were expressed as the mean ± standard deviation. Nominal variables were expressed as percentages. Differences before and after chemotherapy were determined using paired sample t test for continuous variables and Fisher’s exact test for categorical data. Inter- and intra-observer reproducibility of LAV-max, LAV-min, LAVpre-a were assessed using intraclass correlation coefficients (ICCs). Data were analyzed by SPSS version 16.0 (SPSS, Inc, Chicago, IL, USA). A value of p ≤ 0.05 was considered significant.

## Results

### Baseline Characteristics of Patients

Four patients were excluded from the analysis because of poor image that can not be recognized by the workstation. One died before completion of chemotherapy due to pneumonia and secondary respiratory failure. A total of 61 patients, 34 males, ranging from 20 to 70 years (mean age 44.9 ± 11.9 years) were finally include in the statistical analysis. Cumulative dose of doxorubicin was 258.20 ± 69.03 mg/m^2^ (ranging from 200 to 400 mg/m^2^). After treatment of R-CHOP, no patient complained of cardiovascular related symptoms. EKG remained normal in all patients.

### Blood pressure, heart rate, two-dimensional echocardiographical parameters and Cardiac troponin T level measurements

Table 1 summarize the blood pressure, heart rate, two-dimensional echocardiographic findings and cardiac troponin T level measurements in patients before and after chemotherapy.

Systolic blood pressure and diastolic blood pressure were within normal limits for all patients before and after chemotherapy (118.5 ± 9.5 mm Hg vs. 115.6 ± 13.0 mm Hg, P = 0.07; 76.2 ± 7.0 mm Hg vs. 75.1 ± 7.7 mm Hg, P = 0.304). Heart rate is markably faster than that before treatment (78.4 ± 12.2 vs. 82.6 ± 10.3, P = 0.011).

Diastolic posterior wall thickness showed no significant difference before and after chemotherapy (9.3 ± 0.9 mm vs. 9.2 ± 1.0 mm, P = 0.321). Conventional parameters of systolic and diastolic function, including EDV, LVEF, fraction shortening, E velocity, A velocity, E/A ratio, deceleration time, isovolumic relaxation time, average of peak early diastolic velocities (e’), average of peak late diastolic velocities (a’), and E/e’ ratio showed no significant difference after doxorubicin treatment. GLS was significantly reduced after the completion of chemotherapy from −21.64 ± 2.83 to −17.30 ± 2.50, *P* < 0.001.

Before doxorubicin exposure, cTnT levels of 13 patients (21.31%) were below the limitation of detection (<0.003 ng/mL). In these participants, after the completion of chemotherapy, cTnT levels became detectable in the whole cohort. In the analysis, undetectable cTnT levels were considered to be 0.0015 ng/mL^15^,^16^. After the completion of chemotherapy, cTnT levels rose from 0.005 ± 0.004 to 0.020 ± 0.026 ng/mL (*P* all  < 0.001).

### Left atrial volume and function

**Figure 2:**
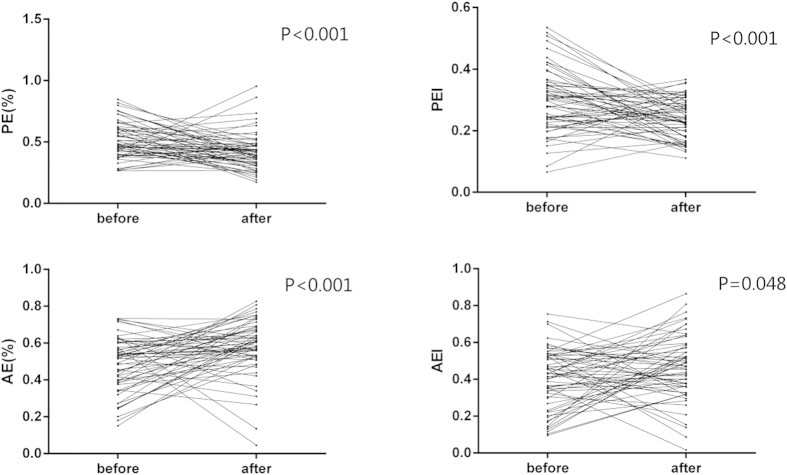
PE, PEI, AE and AEI before and after chemotherapy.

Table 2 depicts various LA volume parameters obtained by RT3DE. Maximal or minimal LA volume index, LA volume index before atrial contraction, filling volume, expansion index and diastolic emptying index showed no significantly difference before and after chemotherapy. Passive emptying percent of total emptying and passive emptying index were significantly reduced after chemotherapy from 0.51 ± 0.14 to 0.40 ± 0.12 (*P* < 0.001), 0.29 ± 0.10 to 0.23 ± 0.06 (*P* < 0.001), respectively. Active emptying percent of total emptying and active emptying index (0.49 ± 0.14 vs. 0.60 ± 0.12, *P* < 0.001; 0.41 ± 0.16 vs. 0.47 ± 0.16, *P* = 0.048) were markedly higher than that value before treatment (Fig. 2).

### Intraobserver and interobserver variability

**Figure 3:**
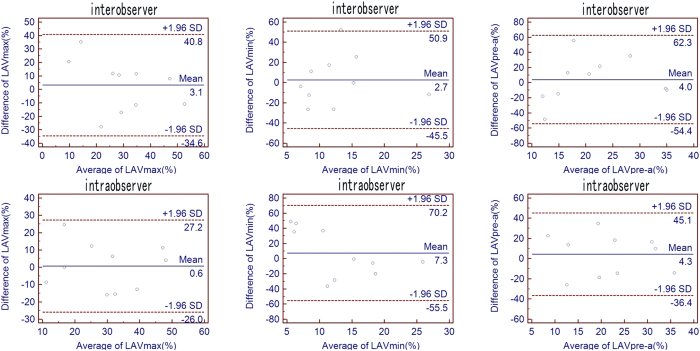
Bland-Altman analysis for intra-observer and inter-observer reliability.

Interobserver measurement showed ICC = 0.94 for LAVmax, 0.86 fore LAVmin, 0.81 for LAVpre-a. Similarly, intraobserver measurement showed ICC = 0.954 for LAVmax, 0.90 for LAVmin, 0.90 for LAVpre-a. Interobserver and intraobserver agreement showed in Fig. 3.

## Discussion

Accurate assessment of LA size should be established by volume measurement. In this respect, RT3DE is a suitable modality. Several studies have reported its feasibility and accuracy for measuring LA volume. With the advantage of time saving and low interobserver variability and its values are comparable to MRI, RT3DE, allowing comprehensive assessment of LA three dimensional structure, may provide reliable and sensitive parameter for early diastolic dysfunction detection. This study showed feasibility and reproducibility of RT3DE in detection of early change in LA function in patients treated with anthracyclines.

It has been suggested that diastolic changes early in various conditions and predict later deterioration. Several studies have reported LV diastolic abnormalities late after anthracycline administration; these abnormalities were assessed by isovolumic relaxation time or transmitral E/A ratio^4^,^5^. In a cross-sectional study, Bu’Lock FA^17^ found that compared with paired control data matched for body surface area, significant abnormalities of diastolic function were present in patients surviving between 6.5 months and 17 years (median 5.3 year) from initial anthracycline treatment for childhood malignancy. However, there was a limited study demonstrating early diastolic dysfunction in patients receiving anthracycline. Importantly, our non-cross-sectional study showed evidence of impaired RT3DE-derived LA function shortly after doxorubicin administration. In our study, diastolic doppler parameters were not significant which was discordant with previous studies, however these studies’ subjects were long-term survivors of childhood cancer. Use the E/e’ ratio remains questionable in the oncological setting, as E and e’ velocities fluctuation in these patients could be the consequence of changes in loading conditions as a results of side effects associated with the chemotherapy (nausea, vomiting, and diarrhea) more than the results of a real change in LV diastolic performance^18^.

Bu’Lock FA’s study also showed that the rate and time of deceleration from peak E velocity were reduced, peak A velocity increased and E/A ratio reduced which were discordant with our results, however, their patients had received anthracycline 401–500 mg/m^2^ which was higher than our cumulative dose^17^.

Our previous study have found that global longitudinal strain could predict cardiac dysfunction in patients receiving anthracycline-based chemotherapy^19^. Thus, early detection of cardiotoxicity will enable to balance between the cardiac risk and the potential cancer treatment benefits in an individual patient. The principal role of the left atrium (LA) is to modulate left ventricular filling and cardiovascular performance by functioning as a reservoir for pulmonary venous return during systole, a conduit for pulmonary venous return during early ventricular diastole and a booster pump that augments ventricular filling during late ventricular diastole^20^,^21^. However, in clinical practice, quantification of LA function remains challenging. Echocardiography is the most commonly used non-invasive imaging technique for estimation of LA size. The M-mode measurement of the LA anteroposterior dimension as indicator for size has several limitations due to geometric assumption made about LA shape and due to slightly diverging position and orientation of imaging planes^22^. It has been suggested that LA volume (LAV) may be a superior index for LA size^23^. Two-dimensional echocardiography derived LAV has been shown to provide a more accurate assessment of LA size than M-mode but the problem of geometric assumption still remains^8^,^24^. RT3DE has provided information about real time changes of LA volumes during cardiac cycle. In fact, there is no research focusing on left atrial volume and function after chemotherapy using RT3DE.

Assessment of the diastolic function has a key role in our understanding of the physiologic damage caused by anthracyclines. To detect the anthracycline-induced cardiotoxicity, assessment of diastolic function is being recommended in addition to systolic parameters, since the evaluation of the E/A ratio alone did not appear enough^17^,^25^. An enlargement of the left atrium reflects the history of chronically increased left atrial pressures. Because the LA volume reflects a “chronic” exposure to abnormal LV diastolic function, the period of our follow-up was not enough to permit the atrium to become extremely dilated^26^. Accordingly, phasic LA volulme was evaluated in patients treated with anthracycline-based chemotherapy using RT3DE and various LA functional parameters obtained by the reconstruction of respective LA time-volume curves. Our study showed that low dose anthracycline significantly affected the indices for LA conduit and booster function, which can be easily obtained by RT3DE. Notably, the interplay between these atrial functions and ventricular performance throughout the cardiac cycle is crucial in many pathophysiological conditions. An interesting finding of our investigation is that, increase in AE and AEI early after anthracycline treatment were accompanied with decrease in GLS and preserved LVEF. Although the median follow-up in our series may still be considered relatively short to assess the diastolic function change in terms of dilated left atrium, our findings suggest that the enhanced booster pump function early after anthracycline treatment may help reduce the left ventricular filling pressure caused by impaired GLS. Therefore, the phasic LA volume may provide a earlier expression of anthracycline-induced subclinical cardiotoxicity and that appeared to be a useful index of cardiovascular risk.

In the study of Cong J *et al*. PEI slightly reduced and AEI mildly increased during the early pregnancy. In the setting of continued volume and pressure overload, significant decreased PEI and AEI were found in in preeclamptic pregnancy^27^. It has been proved that significant abnormalities of left ventricular diastolic filling patterns are associated with prior anthracycline treatment in previous studies. Diastolic filling is affected by hemodynamic loading conditions^28^,^29^,^30^ and particularly by heart rate^31^,^32^ and it might be suggested that the filling abnormalities simply reflect increased heart rate. In our study, heart rate increased significantly after even low dose of anthracycline treatment, which was accordant with Bu’Lock FA’s study. They demonstrated that the heart rate increased in those treated at doses >200 mg/m2^17^. And they also proved that increased resting heart rate was clearly related to treatment intensity and was most apparent at the highest doses and lowest shortening fraction. Hence, it was believed that different patients, chemotherapy regimen and power of study may account for the discordant results. The marked increase of heart rate after treatment may associate with the LA conduit and booster pump function change. Of note, elevated heart rate is likely to reflect systemic effects of chemotherapy agents, volume shifts and proinflammatory state, long-term large–scale study is required to evaluate it.

It has been proposed that abnormalities of Doppler echocardiographic patterns of LV filling change in accordance with the relative degrees of abnormalities of active myocardial relaxation and passive LV compliance^33^. It is therefore possible to postulate some correlations between the LA function change in our study and the underlying anthracycline induced myocardial damage, which although not directly examined in these patients, is well documented in other series^34^,^35^. It might be suggested that in milder degrees of damage associated with sarcoplasmic vacuolation and myofibrillar loss in affected myocytes normal LV systolic function is maintained by enhanced booster pump function of LA.

Preload and afterload is likely to be normal in these patients, which are reflected by normal EDV, PWT and blood pressure. This may also explain the no significant difference of LAVmax, LAVmin and LAVpre-a.

We also found that though the mean cumulative dose of doxorubicin in our study was only 258.20 ± 69.03 mg/m^2^ (ranging from 200 to 400 mg/m^2^), quite lower than the recommended maximum lift time cumulative dose^36^, subclinical cardiac diastolic dysfunction was still observed in these patients, in accordance with the demonstration that anthracycline damage to all cardiac structures may begin with the first anthracycline dose.

Cardiac troponin T is the preferred biomarker for the measurement of myocardial injury. It has been demonstrated that low level elevations of cTnT induced by doxorubicin are associated with histological evidence of myocardial injury and are clinically meaningful^37^. However, the low prevalence of detection with standard assays would limit the utility of troponin measurement for clinical applications in patients after anthracycline exposure. In our study, we analyzed cTnT with a recently developed highly sensitive assay, measuring levels ~10-fold lower than those detectable with the standard assay. We found elevation in high-sensitive cTnT levels after exposure to anthracycline, and the combination of GLS, cTnT, and phasic LAV parameters might be helpful to monitor or earlier detection of anthracycline-induced cardiotoxiciy.

### Limitations of the study

Several limitations to this study warrant comment. The present study does not provide information on the value of LA volume and function parameters in prognostication. Further out-come studies with hard clinical end points will be required to determine the clinical significance of our findings. Secondly, we used a semiautomated contour-tracing software which was designed for LV volume analysis to obtain LA volumes and volumetric curvesm as a results of lacking dedicated atrial software. Thirdly, we did not include LA appendage and pulmonary veins for calculation of LAV and function. Their variability in shape, its difficulty to measure and the lack for standard figures for its normal volume seems reasonable to exclude it. Finally, there was no normal control group, we analyzed the patients parameters by own control comparison.

## Conclusions

Early LA functional change occur after doxorubicin exposure in patients with preserved LVEF, which could be detected by RT-3DE. Phasic LA volumes and parameters might provide a earlier expression of anthracycline-induced subclinical cardiotoxicity.

## Additional Information

**How to cite this article**: Shi, J. *et al*. Early change in left atrial function in patients treated with anthracyclines assessed by real-time three-dimensional echocardiography. *Sci. Rep*. **6**, 25512; doi: 10.1038/srep25512 (2016).

## Figures and Tables

**Table 1:** Vital signs, two-dimensional echocardiographical parameters and cardiac troponin T levels before and after chemotherapy.

	Before	After	*P*
HR	78.4 ± 12.2	82.6 ± 10.3	0.011
SBP	118.5 ± 9.5	115.6 ± 13.0	0.070
DBP	76.2 ± 7.0	75.1 ± 7.7	0.304
EDV	76.4 ± 15.0	77.6 ± 14.7	0.321
PWT	9.3 ± 0.9	9.2 ± 1.0	0.379
LVEF	66.1 ± 4.7%	65.0 ± 3.4%	0.110
FS	38.6 ± 5.1%	39.9 ± 5.1%	0.139
E	69.5 ± 15.0	66.6 ± 15.3	0.085
A	72.0 ± 13.7	69.2 ± 15.5	0.066
E/A	1.0 ± 0.4	1.0 ± 0.3	0.323
DT	181.5 ± 34.0	185.9 ± 33.4	0.407
IVRT	75.0 ± 11.2	71.9 ± 9.9	0.112
e’	12.5 ± 4.3	11.9 ± 3.9	0.237
a’	12.3 ± 3.1	21.0 ± 3.2	0.424
E/e’	6.2 ± 1.9	5.9 ± 1.5	0.302
GLS (%)	−21.64 ± 2.83	−17.30 ± 2.50	<0.001
cTnT (ng/mL)	0.005 ± 0.004	0.020 ± 0.026	<0.001

HR, heart rate; SBP, systolic blood pressure; DBP, diastolic blood pressure; EDV, end-diastolic volume; PWT, posterior wall thickness; LVEF, left ventricular ejection fraction; FS, fraction shortening; DT, deceleration time; IVRT, isovolumic relaxation time; GLS, global longitudinal strain, cTnT, cardiac troponin T level.

**Table 2:** values of LAV and parameters of LA function before and after chemotherapy.

	Before	After	*P*
LAVmax (ml/m^2^)	16.3 ± 5.2	15.7 ± 4.9	0.443
LAVmin (ml/m^2^)	6.8 ± 2.9	6.2 ± 2.7	0.219
LAVpre-A (ml/m^2^)	11.5 ± 4.1	12.0 ± 4.3	0.400
Filling volume (ml)	9.49 ± 4.15	9.43 ± 3.70	0.925
Expansion index (%)	1.69 ± 1.15	1.84 ± 1.34	0.511
DEI	0.58 ± 0.14	0.60 ± 0.13	0.426
PE (%)	0.51 ± 0.14	0.40 ± 0.12	<0.001
PEI	0.29 ± 0.10	0.23 ± 0.06	<0.001
AE (%)	0.49 ± 0.14	0.60 ± 0.12	<0.001
AEI	0.41 ± 0.16	0.47 ± 0.16	0.048

AE, Active emptying % of total emptying; AEI, active emptying index; DEI, diastolic emptying index; LAVmax, maximum left atrial volume index; LAVmin, minimum left atrial volume index; LAVpre-a, left atrial volume index before atrial contraction; PE, passive emptying % of total emptying; PEI, passive emptying index.
